# Deciphering strain differences in codY regulation of *Clostridioides difficile*s sporulation

**DOI:** 10.1128/spectrum.01706-25

**Published:** 2025-11-17

**Authors:** Marcos P. Monteiro, Adrianne N. Edwards, Michael A. DiCandia, Shonna M. McBride

**Affiliations:** 1 Department of Microbiology and Immunology, Emory University School of Medicine, Emory Antibiotic Resistance Center 12239, Atlanta, Georgia, USA; 2 Center for Integrated Solutions for Infectious Diseases, Broad Institute of MIT and Harvard, Cambridge, Massachusetts, USA; The Pennsylvania State University, University Park, Pennsylvania, USA

**Keywords:** *Clostridium difficile*, nutrient availability, CodY, sporulation

## Abstract

*Clostridioides difficile* is an anaerobic, spore-forming pathogen that causes diarrhea, colitis, and even death. *C. difficile* grows and replicates in the intestine as a vegetative bacillus, but must transition into a dormant spore to survive and transmit in the environment. The transformation into a spore is a complex developmental process that is regulated in response to conditions within the host, most notably nutrient limitation. Nutrient availability is sensed by *C. difficile* through transcriptional regulators, such as CodY. CodY is a global nutritional gene regulator that controls gene expression in response to branched-chain amino acids (BCAAs) and guanosine-triphosphate (GTP). It was previously observed that CodY represses *C. difficile* sporulation, but the impact of CodY on sporulation has differed considerably by strain. Here, we investigated the effects of CodY on gene expression during sporulation in the two common research strains 630*∆erm* (ribotype 012) and UK1 (ribotype 027). We confirmed that CodY suppressed premature spore formation in both strains through time-elapsed sporulation assays with *codY* mutants. Through transcriptional analyses of *codY* mutant sporulation, we defined the similarities and differences in the CodY-dependent gene expression between strains. We also identified differences in putative CodY sites within the 630 and UK1 genomes that may influence CodY regulation. Finally, we performed CRISPRi knockdowns to examine the effects of selected CodY-regulated genes, demonstrating the impact of multiple CodY-dependent factors on sporulation.

**IMPORTANCE:**

*Clostridioides difficile* spore formation is crucial for transmission and survival of the bacterium. Spore formation is triggered by the availability of crucial nutrients, which CodY and other regulators sense. However, the mechanism by which CodY represses sporulation in *C. difficile* is poorly understood. In this study, we identified several CodY-regulated factors that could play a role in sporulation both in 630*∆erm* and UK1 strains. Our results show that many factors under the regulation of CodY can impact sporulation.

## INTRODUCTION

*Clostridioides difficile* is an anaerobic and spore-forming nosocomial pathogen that causes severe diarrhea, colitis, and even death (1–3). Transmission of *C. difficile* is only possible through spores, which survive environmental threats, such as atmospheric oxygen and disinfectants (4). After a host ingests *C. difficile* spores, they transit through the gastrointestinal tract, reaching the intestines, where they sense bile salts and germinate into vegetative cells (5–7). *C. difficile* vegetative cells colonize the host colon, where nutrient availability is limited, leading to toxin production and spore formation (8–14). Nutrient availability is fundamental for determining whether *C. difficile* grows as a vegetative cell or becomes a spore. Under nutrient-limited conditions, *C. difficile* responds by increasing the expression of factors for nutrient acquisition and biosynthesis of necessary metabolites; when these mechanisms fail to provide for sustained vegetative growth, spore formation is initiated (15–17).

To sense and control metabolism, *C. difficile* encodes nutritional regulators, such as the global nutrient transcriptional regulator, CodY (11, 13, 14, 18). CodY was first identified in *Bacillus subtilis* and is present in many gram-positive bacteria with low G-C genomes (19–26). In a nutrient-rich environment, *C. difficile* senses branched-chain amino acids (BCAAs) and guanosine triphosphate (GTP) through their interactions with CodY (11, 13, 14, 27, 28). CodY undergoes a conformational change when it binds to BCAAs and GTP, which increases its binding affinity to specific CodY-DNA binding sites, leading to the differential regulation of hundreds of genes (11, 13, 14, 29). When the intracellular concentrations of BCAAs and GTP decrease, the binding affinity of CodY to DNA is altered, changing gene expression to adapt to nutrient scarcity, including the derepression of toxin production and the initiation of sporulation (11, 13, 14, 29–31). While the regulation of specific metabolic genes and toxins by CodY is well-documented, the mechanisms by which CodY affects *C. difficile* sporulation are less clear (29, 32). CodY has varied effects on sporulation in strains 630 (ribotype 012) and UK1 (ribotype 027), as evidenced by a modest increase in sporulation in a 630 *codY* mutant and robust hypersporulation in a UK1 *codY* mutant (13, 29). The CodY proteins encoded by these strains are identical and similarly expressed, leading us to ask how CodY differentially regulates sporulation outcomes in these strains.

In this study, we examined CodY-dependent gene regulation in the 630 and UK1 backgrounds to identify strain-specific differences in sporulation outcomes. Through transcriptional analysis and mapping of CodY-binding sites, we identified CodY-regulated factors that are differentially expressed in 630*∆erm* and UK1 and contain a CodY-binding site in at least one strain. In addition, we demonstrated that transcriptional repression of several direct CodY-regulated factors in UK1 or UK1 *codY* impacts sporulation. These results illustrate how CodY regulation differs between the 630*∆erm* and UK1 strains and demonstrate that many CodY-regulated factors can impact sporulation.

## RESULTS

### The impact of CodY regulation on sporulation is strain-dependent

**Fig 1:**
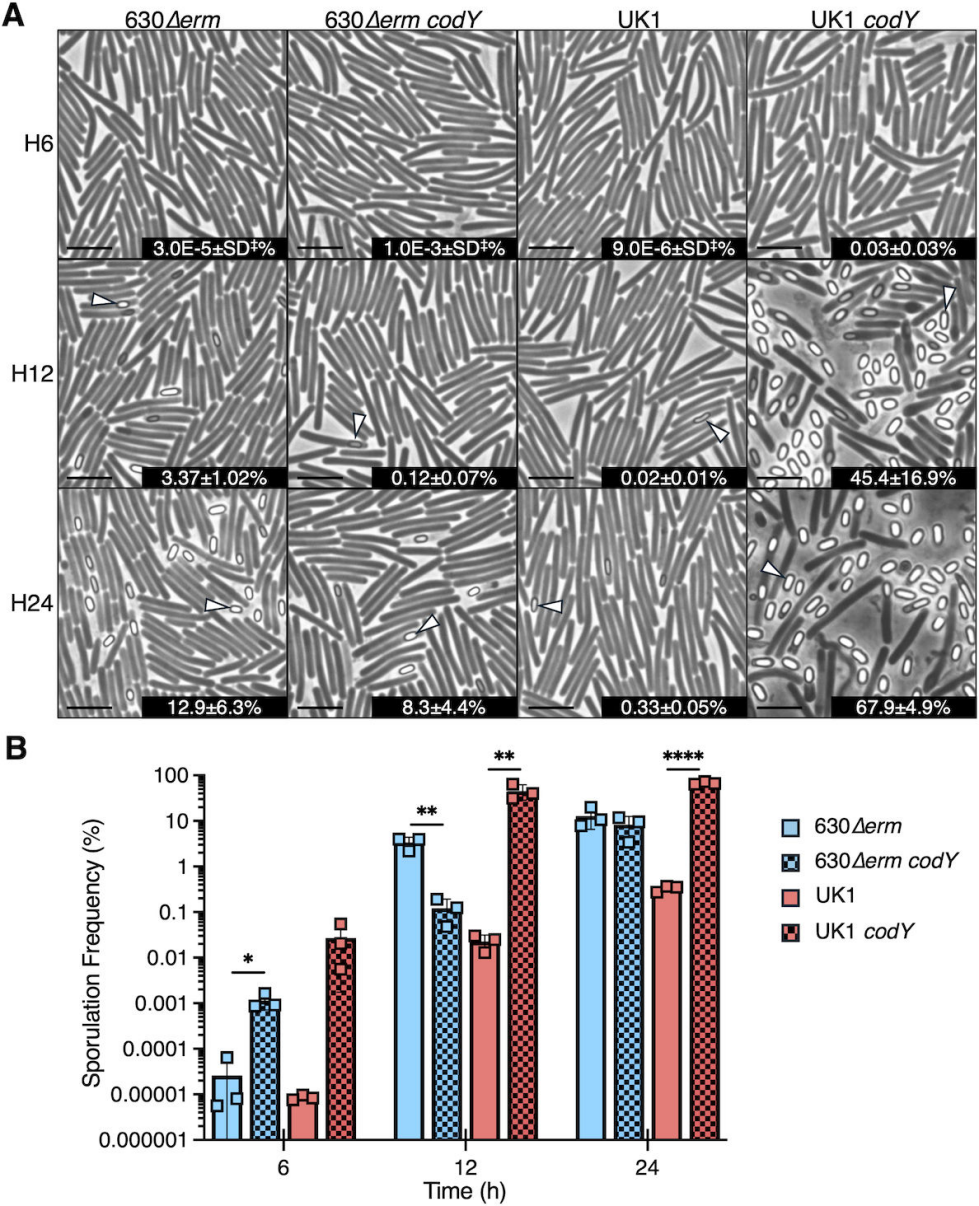
CodY repression on sporulation is strain-dependent. (**A**) Phase-contrast micrographs of strains 630*∆erm*, 630*∆erm codY* (MC364), UK1, and UK1 *codY* (LB-CD16) grown on sporulation agar for 6, 12, or 24 h. White arrowheads indicate bright spores. Scale bar = 5 µm. ^‡^SD: standard deviation <0.0001. (**B**) Ethanol-resistant spore formation for the cultures above. The means and individual values for three biological replicates are shown. Data were analyzed using unpaired Student’s *t*-tests comparing the mutants to their respective parent strain. *P < 0.05, ** *P* < 0.01, and **** *P* < 0.0001.

In a previous work, we demonstrated that CodY represses the initiation of sporulation and that CodY regulation of sporulation varies by strain (13). In the commonly studied strain 630*∆erm* (a 630 derivative), CodY was found to modestly repress sporulation, resulting in a twofold increase in sporulation frequency for the *codY* mutant in sporulation broth cultures. In contrast, the epidemic 027 isolate, the UK1 *codY* mutant, demonstrated more than 1,000-fold greater sporulation frequency than the parent strain. To better understand how CodY regulates sporulation dissimilarly in 630*∆erm* and UK1, we evaluated sporulation in these strains over time on sporulation agar, which induces more robust sporulation than liquid medium (33, 34). Strains UK1, 630*∆erm*, and their respective *codY* mutants were grown on 70:30 sporulation agar, and the formation of ethanol-resistant spores was assessed after 6 (logarithmic phase), 12 (stationary phase), and 24 h of growth to compare the dynamics of spore production. As shown in Fig. 1, at log phase, the 630*∆erm codY* mutant sporulated ~43-fold more than its parent strain (1.0E−3 ± 4.3E−4 vs 2.6E−5 ± 3.3E−5%). In comparison, at log phase, the UK1 *codY* mutant sporulated ~3,150-fold more than its parent strain, UK1. These results support the prior evidence that CodY represses premature sporulation initiation and that CodY repression of sporulation in UK1 is more robust than in 630*∆erm* (13). By stationary phase (12 h), the 630 *codY* mutant sporulated ~28-fold less than the parent strain (0.12 ± 0.07 vs 3.37 ± 1.02%), while after 24 h of growth, the 630 *codY* mutant and parent displayed similar sporulation frequencies (Fig. 1). These results suggest that CodY suppresses early initiation of sporulation in 630 and that this strain requires CodY to control the timing of sporulation. In contrast, at stationary phase, the UK1 *codY* mutant sporulation frequency was ~2,000-fold higher than its parent strain (45.4 ± 16.9 vs 0.02 ± 0.01%) and continued at greater frequency than the parent at 24 h (67.9 ± 4.9 vs 0.33 ± 0.05). Thus, the UK1 strain CodY represses sporulation at all growth stages. These data suggest there are differences in CodY-dependent gene regulation in U K1 and 630 that result in dissimilar sporulation outcomes.

### Identifying strain-specific differences in CodY regulation

To understand how CodY regulates sporulation differently in the UK1 and 630 backgrounds, we examined gene expression during growth on sporulation agar in these strains and their *codY* mutants. Since CodY activity is controlled by the availability of BCAA and GTP, we investigated expression at log phase when nutrients are most abundant, and CodY repression is greatest (11, 13, 14, 19, 27–29, 35, 36). Following 6 h of growth on 70:30 agar, samples were processed for RNA-seq analysis to assess the ratio of gene expression in the *codY* mutants relative to their respective parent strain (*codY*/WT) (Tables S1 and S2). Transcription was extensively altered in the *codY* mutants of both strains, resulting in 867 genes differentially expressed more than threefold in the UK1 *codY* mutant and 449 genes in the 630 *codY* mutant.

**Fig 2:**
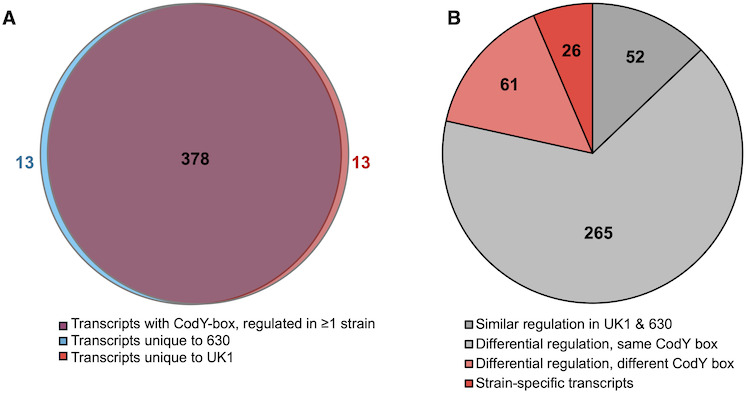
Representation of CodY-regulated transcripts with putative CodY boxes in strains UK1 and 630. (**A**) Venn diagram illustration of CodY-regulated genes and operons from RNA-seq data of strains UK1 and 630 that contain predicted CodY-box regulatory elements. A total of 404 transcripts that were differentially regulated ≥3-fold in UK1 ∆*codY*, 630∆*erm* ∆*codY,* relative to their parent strains, are represented. (**B**) Representation of CodY-regulated transcripts from A categorized by regulation characteristics.

Transcripts that were differentially regulated in the UK1 *codY* and 630 *codY* mutants include factors that are directly and indirectly regulated by CodY. To discern which genes may be directly controlled by CodY to influence sporulation, we sought to define genes with CodY-binding motifs (CodY boxes). Using the CodY binding sites previously identified in *C. difficile* (13, 14, 37) and potential CodY boxes identified based on the classical gram-positive CodY consensus (AATTTTCWGAAAATT) (38, 39), we narrowed the list of differentially regulated genes to those most likely to be directly regulated by CodY. The resulting list included 404 transcripts with prospective CodY-binding sites within the promoter or coding sequence that were threefold differentially expressed in at least one of the *codY* mutant strains relative to the parental control (Fig. 2; Table S3).

Of the genes and operons listed in Table S3, 52 were similarly regulated by CodY in the UK1 and 630∆*erm* strains, which limits their likelihood for strain-specific, CodY-dependent impacts. While some of these factors may differ in protein similarity or function that results in differences in sporulation outcomes, such differences were outside the scope of this study. Of the 352 transcripts in Table S3 that were dissimilarly CodY regulated between UK1 and 630∆*erm*, 265 had identical CodY boxes, which suggests that the differences in expression observed were not due to variation in the inherent ability of CodY to bind to these target sequences. We focused further on the 87 CodY-regulated transcripts with significant differences in expression between the UK1 and 630∆*erm* strains (Fig. 2B and Tables 1 and 2). Table 1 includes CodY-regulated genes with associated CodY boxes that differ in expression at least twofold between strains, while Table 2 contains CodY-regulated genes that are unique to the genome of either strain. As expected from the sporulation phenotypes of the *codY* mutants, sporulation-specific transcripts comprised many of the genes differentially expressed in the UK1 *codY* mutant (Table S3 ~10%) (33, 40), many of which were late-stage sporulation or germination factors. Unfortunately, increased late sporulation gene expression in UK1 *codY* is not helpful for understanding how CodY differentially regulates the initiation of sporulation, which is controlled by the activation of the master sporulation regulator, Spo0A (33, 41). One factor that is directly involved in Spo0A activity and demonstrated reduced expression in UK1 *codY* is *spo0E*. Spo0E interacts with Spo0A to limit Spo0A activation, which prevents sporulation initiation in *C. difficile* (42). However, the putative CodY boxes that potentially impact *spo0E* were identical in UK1 and 630, implying that the CodY-dependent effect on *spo0E* transcription in the UK1 *codY* mutant was not due to strain-specificity in CodY binding. In addition, a large proportion (20%, Table S3) of the CodY-regulated transcripts in both strains are genes of unknown function, which limits our understanding of their contribution to CodY-dependent phenotypes.

**TABLE 1:** Differentially expressed CodY target genes in strains 630*∆erm* and UK1

UK1				630∆*erm*					
Genetic region	Predicted CodY box^*a*^	Predicted CodY target	∆codY/WT	Genetic region	Predicted CodY box	Predicted CodY target	∆*codY*/WT	Gene names	Putative function
CDIF27147_00336-00337	AATATTCAAATAATT AACTTTAGGAAAAAT AATTTTTTGAAAAAA	CDIF27147_00336-00337	16.5–18.3	CD02130-02140	AATATTCAAATAATT AACTTTA**A**GAAAAAT AATTTTTTGAAAAAA	CD02130-02140	1.43–1.77		Sporulation
CDIF27147_00351-00353	AATTTTCTGACAAAT	CDIF27147_00352-00353	0.23–0.28	CD02260-02280	A**G**TTTTCTGACAGCT	CD02270-02280	1.57–4.41	*fliN*	Motility
CDIF27147_00374-00397	AACTTTTAGAAAATA AAGTTTATGAAAATT AATTTTGAGAAAAAT	CDIF27147_00382-00397	0.39–0.95	CD02450-02630	AACTTTT**G**GAA**G**ATA AAGTTTATGAAAATT AATTTTGAGAAAAAT	CD02670	1.21–4.13	*flg, fli, mot, flh*	Motility
CDIF27147_00476-00478	CATTTTAGAAAAATT	CDIF27147_00478	1.30–3.28	CD03350-03370	CATTTTA**A**AAAAATT	CD03370	0.59–8.06		Unknown
CDIF27147_00481-00482	AAATATCTGAAAAAA AATTTACTAAAAACT AAGTTTATGAAAAAT GATTTTATGCAAATT	CDIF27147_00481-00482	0.24–0.27	CD03400-03410	AA**T**TATCTGAAAAAA AATTTACTAAAAACT AAGTTTATGAAAAAT GATTTTATGCAAATT	CD03400-03410	1.43–1.62		Unknown
CDIF27147_00526-00530	AAAATTCGGAAAATT	CDIF27147_00526-00530	10.05–23.88	CD04450-04490	AAAATTC**A**GAAAATT	CD04450-04490	0.93–1.34	*oraSE, orr*	Amino acid
CDIF27147_00566-00567	AGATTTGTGAAAATA AATTTTGAAAATAGT AATATTTTAAAAATC ATCTTTCTCACAATT AAGTTTCAAGAAATA AACTTACTAAAAATC	CDIF27147_00566	1.12–6.02	CD04830-04840	AGATTTGTGAAAATA AATTTTGAAAATAGT AATATTTTAAAAATC ATCTTTCTCACAATT AAGTTTCAAGAAATA AACT**C**ACTAAAAATC	CD04830	1.38–1.45		Transporter
CDIF27147_00618	AACATTCTGAAAAAT AATATAACGAAAATT AAGTTAAAGAAAATT	CDIF27147_00618	8.64	CD05500	AA**T**ATTCTGAAAAAT AATATAACGAAAATT AAGTTAAAGAAAATT	CD05500	1.08		Unknown
CDIF27147_00723-00725	AATATACTTTAAATT AACCTTTATAAAATT AGTTTGAAAAAAATT **AAATATCTGTATATT** AATTATATCAAAATC GATTTTATGGAATTT AATTTACAGATACTG AAAGTTCAGATATTT AGATTTATGAAGATT AATTTGCAGAACTAT CATTACCTGAAAAAT AAATATGTGAAAAAT AACTTTCAGAGATTA	CDIF27147_00723-00725	2.10–4.13	CD06490-06510	AATATACTTTAAATT AACCTTTATAAAATT AGTTTGAAAAAAATT AATTATATCAAAATC GATTTTATGGAATTT AATTTACAGATACTG AAAGTTCAGATATTT AGATTTATGAAGATT AATTTGCAGAACTAT CATTACCTGAAAAAT AAATATGTGAAAAAT AACTTTCAGAGATTA	CD06490-06510	1.01–1.10		Peptidases
CDIF27147_00734-00738	AATGTTGTCAAAATT AATTTGATGAAAATA AATTTTTAAATAAAT AAGTTACAGAAAATA **ACTTTTCTCAAAATA** AATTCCCAGAAAATA TATTTTCTAAAAATC AATTTTTTCAAAATA GATTTTGTGAAAAAT AAGTTTCTGGAAATT	CDIF27147_00734-00738	49.0–259.4	CD06590-06630	AATGTTGTCAAAATT AATTTGATGAAAATA AATTTT**C**AAATAAAT AA**A**TTACA**C**A**G**AATA AATTCCCAGAAAATA TATTTTCTAAAAATC AATTTTTTCAAAATA GATTTTGTGAAAAAT AAGTTTCTGGAAATT	CD06590-06630	5.02–436.3	*tcdRBEA*	Toxin
CDIF27147_00739	TATTTTAGGAAAATA	CDIF27147_00739	33.2	CD06640	TATTTT**CCT**AAAATA	CD06640	5.81	*tcdC*	Toxin
CDIF27147_00772	AATTATAAGAAGATT	CDIF27147_00772	10.3	CD06910	A**G**TTATAAGAAGATT	CD06910	0.43		Metabolism
CDIF27147_00939-00943	AATTTGATGAAATTT AATTTTTAAAAAGTT AATTTACGGCAAATG	CDIF27147_00939-00943	0.23–0.53	CD08530-08560	AATTTGATGAAATTT AATTTTTAAAAAGTT AATTTAC**A**GCAAATG	CD08530	0.58–1.12	*oppBCAD*	Metabolite transporter
CDIF27147_00947-00949	TATTATCTGAAAATA AAGTTTTAGAAATTT	CDIF27147_00948-00949	1.23–2.12	CD08610-08630	TATTATCTGAAAATA AAGTTTTAGAAA**C**TT	CD08620-08630	0.27–0.79		Metabolite transporter
CDIF27147_00969-00973	AATTTTATGAAAGCT TATTTTTAGAGAATT AATTTCCTCAAAAGT	CDIF27147_00969-00973	4.92–6.74	CD08820-08860	AATTTTATGAAAGCT TATTTTTAGAGAATT AATTTCCTCAAAA**A**T	CD08820-08860	1.65–2.48	*glgCDAP*	Metabolism
CDIF27147_01044-01045	CTTTTTTAGAAAATT ATTTTTATGAGAATT AATTTTAAGAATATA AAGTTTATTAAAATT AATATTAGTAAAATT	CDIF27147_01044-01045	3.67–20.0	CD10280-10290	CTTTTTTAGAAAATT ATTTTTATGAGAATT AATTTTAAGAATATA AAGT**C**TATTAAAATT AATATTAGTAAA**G**TT	CD10280-10290	0.49–1.06		Signaling
CDIF27147_01075	AATTATTGAAAAATT AAATTTCACAAAATT TATTTCAGGAAAATT	CDIF27147_01075	0.23	CD10540	AATTATTGAAAAATT AAATTTCACAAAATT TATTTCAGGAAAA**C**T	CD10540	0.5	*bcd2*	Metabolism
CDIF27147_01249			0.89	CD12380	**AATTTTAGGAACATT**	CD12380	4.19		Unknown
CDIF27147_01280-012820	AATTTTCAGCATATT AGATTTCTCAAAATT AATTTTATAAAAAAT	CDIF27147_01280-012820	0.64–0.80	CD12660-12680	AATTTTCAGTATATT **AATTAAAAGAAAATT AATTCTCAGAAAATA**	CD12660-12670	2.04–3.21		Transporter
CDIF27147_01285-01288	**CAATTTCAAAAAATT** ATTTTTCTGAAAAAG AATATCCTGAAAATT AATTTGGAGAAGATT AATTTCCATAAATTT	CDIF27147_01285-01288	3.36–8.81	CD12710- 12740	ATTTTTCTGAAAAAG AATATCCTGAAAATT AATTTGGAGAAGGTT AATTTCCATAAATTT	CD1273-12740	0.25–1.10	*topA*	DNA processing
CDIF27147_01432	CATTTGAAGAAAATT AATTTTAAGTATATT **AATTTTCTTATATTT**	CDIF27147_01432	0.29	CD14120	CATTTGAAGAAAATT AATTTTAAGTATATT	CD14120	1.16		Transcription regulation
CDIF27147_01501	**ACTTTGCAGAAAGTT**	CDIF27147_01501	0.28	CD14750	**GATATTCAAATAATT**	CD14750	0.54		Unknown
CDIF27147_01665			49.8	CD15670	**AATATTGATAAAATT**	CD15670	1.01	*cotG*	Sporulation
CDIF27147_01721	ATTTTTCAGACAATT AAATTTTACAAAATT AATTTTGCGTAATTT AATTTAACAAAAATT AATTTTATTATAATT	CDIF27147_01721	0.30	CD16160	ATTTTTCAGACAATT AAATTTTACAAAATT AATTTTG**T**GTAATTT AATTTAACAAA**G**ATT AATTTTATTATAATT	CD16160	1.29		Signaling
CDIF27147_01737	AATTATTGCAAAATT	CDIF27147_01737	28.1	CD16310	AATT**T**T**GCA**ATAATT	CD16310	0.59	*sodA*	Metabolism
CDIF27147_01805-01806	AATTTTCTTTAAATT	CDIF27147_01805-01806	0.88–1.67	CD16940-16950	**ATTTTTCAAAAACTT AATTTTTCAAAAACT** AATTTTCTTTAAATT	CD16940-16950	0.23–0.71		Unknown
CDIF27147_01855-01856	AATTATTGGTAAATT	CDIF27147_01855-01856	6.03–6.48	CD17400-17410	AATTATTG**C**TAAATT	CD17400-17410	0.55	*grdGF*	Metabolism
CDIF27147_01886	AATTTTAAAAAAATT **AATTTTTTGAAAAAA** CATTTTCCTAATATT	CDIF27147_01886	0.02	CD17671-17680	AATTTTAAAAAAATT CATTTTCCTAATATT	CD17671-17680	0.09–0.11		Unknown
CDIF27147_01913	AAATTCCTAAAAATT	CDIF27147_01913	4.77	CD17930	AA**G**T**G**CCTAAAAATT	CD17930	0.52		Unknown
CDIF27147_01965	AATTTTACGATATTT ATTTTCGAGAAAAAT	CDIF27147_01965	8.23	CD18440	AATTTTACGATATTT **AAAATACAGAAAATT** ATTTTTGAGAAAAAT	CD18440	1.02		Unknown
CDIF27147_02022-02023	GATTTTCATAACATT **AATTTTCAAAGATTT AAATTTCTAAAAATG**	CDIF27147_02022-02023	4.15–6.48	CD18620-18630	GATTTTCATAACATT	CD18620-18630	0.35–0.58		Conjugative Transposon
CDIF27147_02031	AACTTTCAGACAAAT	CDIF27147_02031	0.11	CD18710	AACT**C**TCAGACAAAT	CD18710	0.55		Conjugative transposon
CDIF27147_02062-02067	AATTTTTTCTAAATT GATTTGCAGAAAGTT AAGTTTCAGAAGATA	CDIF27147_02062-02067	16.8–22.8	CD19120-19170	AATTTTTTCTAAATT GATTT**A**CAGAAAGTT AAGTTTCAGAAGATA	CD19120-19170	0.18–0.42	*eutABCLME*	Metabolism
CDIF27147_02068	AAATTTATAAAAATA	CDIF27147_02068	67.5	CD19180	AAATTT**C**TAAAAATA	CD19180	0.31	*eutK*	Metabolism
CDIF27147_02170	**ATATTTACGAAAATT**	CDIF27147_02170	321.8	CD20000			0.12	*ispD*	Metabolism
CDIF27147_02368	AATTTTAAGAATATA AAAATTCTGAAATTT	CDIF27147_02368	33.3	CD22010	AATTTT**G**AGAATATA AAAATTCTGAAATTT	CD22010	7.11		Transporter
CDIF27147_02391-02392	ATTATTCAAAAAATT	CDIF27147_02391-02392	2.25–3.91	CD22310-22330	**T**TTATTCAAAAAATT	CD22310-22330	0.95–1.35	*asrABC*	Redox
CDIF27147_02414	GAATTACTAAAAATA AAGCTTGTGAAAAGT AATATTCATAAATGT AATTTATTGTAATTT AATTTTAATAATCTT	CDIF27147_02414	0.64	CD22520	GAATTACT**G**AAAATA AAGCTTGTGAAAAGT AATATTCATAAATGT AATTTATTGTAATTT AATTTTAATAATCTT	CD22520	5.93	*kamA*	Metabolism
CDIF27147_02424	AATATTCTGAAGATA AAATTACAGATAAAT AATCTTTTGAAAAAG ATTTGACTGAAAAAT AAAATTCAGATAATG	CDIF27147_02424	0.06	CD22630	AATA**C**TCTGAAGATA AAATTACAGATAAAT AATCTTTTGAAAAAG ATTTGACTGAAAAAT AAAATTCAGATAATG	CD22630	1.89	*prsA*	Metabolism
CDIF27147_02479-02480	AATTCTATGAAAATT	CDIF27147_02479-02480	0.63–0.75	CD23260-23270	AATTCTAT**A**AAAATT	CD23260-23270	3.89–4.50	*gatAB*	Metabolism
CDIF27147_02545-02546	ATTTTTTAGAAAGTT AATTTAAAAAAAATT	CDIF27147_02545-02546	0.37–0.36	CD23880-23900	ATTTT**C**TAGAAAGTT **AATTTTATGAAGATA** AA**A**TTAA**G**AAAAAT**A**	CD23880-23890	1.18–3.24	*blaRI*	Antimicrobial resistance
CDIF27147_02668	ATTTTTCTGAATATT TATTTTCATAATATT AAATTTCATAAGATT	CDIF27147_02668	2.13	CD25020	ATTTTTCTGAATATT TATTTTCATAATATT AAATTT**T**ATAAGATT	CD25020	6.81		Cofactor synthesis
CDIF27147_02763			48.6	CD25990	**AATTATATTTAAATT**	CD25990	0.50		Transcriptional regulator
CDIF27147_02961	AGTATTCTGAAAGTT	CDIF27147_02961	0.32	CD27870	AGTATTCTGAAAG**C**T	CD27870	0.82	*cwp84*	Cell surface
CDIF27147_02971	AATATTCAGAAAAAA AGTTAGCAGAAGATT AATTTACTGATAGTA TATTGTCTGAAACTT AATATACACAAAATT	CDIF27147_02971	0.33	CD27970	**G**ATATTCAGAAAAAA A**A**TTAGCAGAAGATT AATTTACTGATA**A**TA TATTGTCTGAAACTT AATATACACAAAATT	CD27970	3.54		Cell surface
CDIF27147_02995	**AATTTTAAGAAAGTT** AATTTTCAATAAGTT	CDIF27147_02995	10.2	CD28181 (partial)	AATTTTCAATAAGTT	CD28181	1.09		Unknown
CDIF27147_03022	GATTTTAGGAAAATT AATTCACTGAGAGTT AATTTTCATTTAATT	CDIF27147_03022	78.5	CD28370	GATTTTAGGAAAATT AATT**T**ACTGAGAGTT AATTTTCATTTAATT	CD28370	5.47		Unknown
CDIF27147_03138	AATTTGACAAAAATT AAGTTTGAAAAAAAT TATTATCAGAAAGTT	CDIF27147_03138	0.51	CD30040	A**T**TTTGACAAAAATT AAGTTTGAAAAAA**T**T TATTATCAGAAAGTT	CD30040	4.40	*kdgT2*	Metabolite transport
CDIF27147_03140	AATATTCTGTATATG AAATTAGTGAAAATT	CDIF27147_03140	0.41	CD30060	AATATTCTGTATAT**T** AAATTAGTGAAAATT	CD30060	4.60		Metabolism
CDIF27147_03156-03157	AATGTTCCTAAAAAC ATATTTTAGAAAATT	CDIF27147_03156-03157	101.4–174.3	CD30230-30240	AATGTTCCTAAAAA**T** ATATTTTAGAAAATT	CD30230-30240	0.21–0.37		Unknown
CDIF27147_03165	ATTTTTTATAAAATT AATTCTTTGAAAAAT	CDIF27147_03165	37.1	CD30320	ATTTTTTATAA**G**ATT AATTCTTTGAAAAAT	CD30320	0.93		Cofactor synthesis
CDIF27147_03235-03236	AATTTATTTAGAATT	CDIF27147_03235-03236	1.50–2.46	CD30970-30980	AATTTATTTA**A**AATT	CD30970-30980	5.28–6.89	*bglGF*	Metabolite transport
CDIF27147_03313-03314	AATTATAAGCAAATT	CDIF27147_03313	2.36–9.10	CD31510- 31521	AAT**C**ATAAGCAAATT	CD31510	0.61–1.26		Prophage transcription regulation
CDIF27147_03355	AATATTTATAAAATT	CDIF27147_03355	14.3	CD31840	AA**A**ATTTATAAA**C**TT	CD31840	0.82	*dpaL*	Metabolism
CDIF27147_03396	TATTTTCTAATAATT	CDIF27147_03396	3.83	CD32190	TATTTT**T**TAATAATT	CD32190	0.93	*hslO*	Stress response
CDIF27147_03439-03442	AAAGTACAGGAAATT	CDIF27147_03439-03442	5.71–10.1	CD32600-32630	AAAGTACAGGAAATT **AATTTGATGGAAATA**	CD32600-32630	0.45–1.00	*pstCAB, phoU*	Transporter, transcription regulation
CDIF27147_03542	GATTTTCTGAAAAGA GAATTTCAAAAAAGT	CDIF27147_03542	0.26	CD33690	GATTTTCTGAAAA**A**A **A**AATTTCAAAAAAGT	CD33690	5.63		Unknown

^*a*^ Differences in CodY boxes are noted in bold.

**TABLE 2:** Unique direct CodY-regulated factors present in the 630*∆erm* or UK1 strains

Genetic region	Predicted CodY box	Predicted CodY target	*∆codY*/WT	Gene names	Putative function
630*∆erm*					
CD02110-02120	AATTTGATGAAAATA GATTTTCGGAAAAAT	CD02110-02120	2.08–3.89	*licC*	Metabolism
CD02410-02440	ATTTTTTTTAAAATT TATATTCTAAAAATT GATTTTCTGATAATG	CD02410-02440	4.31–8.55		Motility
CD03790-CD03810	AATATACGGAACATT	CD03790-03810	0.34–0.60		Conjugative transposon
CD04090-04120	AAATTTCATAAAAAT	CD04090-04120	0.33–1.11		Conjugative transposon
CD04230	AATTTTCAAAGACTT AAATTACAGAAAAAT AAATTTCTAAAAATG AATATGCTGAAAATC	CD04230	0.32		DNA replication
CD04352	TACTTTCAGAACATT	CD04352	0.27		Conjugative transposon
CD10921-10940	ACTTTACAGAAGATT	CD10921-10940	0.23–0.27		Conjugative transposon, transcriptional regulator
CD11030	AAGTGTCAGAAAATG	CD11030	0.32		Conjugative transposon
CD18510-18550	GACTTTCTCAAAATT	CD18510-18550	0.30–0.68		Conjugative transposon
CD18840	AATTTTTATAATATT	CD18840	0.07		Unknown
CD18860	AATTTTAGGATTATT AATTTACAGCAACTT	CD18860	4.47		Transcription regulator
CD26170	AATATTCCAAAATTT	CD26170	5.33		Unknown
CD31360-31380	AATTTTATGATGATT ATTTTTATGAAAATT AATTTACTAAAGATT	CD31360-31380	2.95–5.86	*bglA7F5G4*	Metabolism
UK1					
CDIF27147_00347-00350	ATTTTCCTGAAAAAT	CDIF27147_00350	0.23–0.59	*rfbBCAD*	Metabolism
CDIF27147_ 00657-00658	AATTTTCTTAATATT	CDIF27147_00657-00658	9.18		Signaling
CDIF27147_00757	ACTTAACTGAAAATT	CDIF27147_00757	24.0		Amino acid metabolism
CDIF27147_ 01970-01972	AACTTTTGGAAAAGT	CDIF27147_01972	1.49–7.30		Conjugative transposon
CDIF27147_ 02077-02078	AATTTACTAAAAATA AATATTGAGAAAAAT	CDIF27147_02077-02078	1.08–3.09		Metabolism
CDIF27147_03267	AATATTCAGGAACTT	CDIF27147_03267	1.56–3.34		Metabolism
CDIF27147_ 03305-03309	AATTTTTAAAATATT GATTTTATGAAAATA AATGTTAGGAAAATT AATTTATGGAAGATT ACTTTTAGGAAAATA AGTTTTTAGAAACTT ATATTTTAGAAAATT	CDIF27147_03305-03309	0.29–0.50		CRISPR
CDIF27147_03444-03445	AATTTTCTCATAATC	CDIF27147_03444-03445	4.54–5.27		Transporter
CDIF27147_03612	AATTTTCAAAAAGAT AATTTGGAGAAGATT	CDIF27147_03612	0.29		Unknown
CDIF27147_03617	AATTTTCTGATGATG	CDIF27147_03617	4.00		Unknown
CDIF27147_03628	AATTTTTTAAAACTT AATTTTTACAAAAAT	CDIF27147_03628	7.05		Unknown
CDIF27147_03629	AATTTGCAAAAGATT AATTTTTATAAACTT	CDIF27147_03629	10.8		Transposase
CDIF27147_03815-03818	CATTTTTGGAAACTT	CDIF27147_03818	2.31–6.48		Transposon

Though few sporulation initiation-associated genes were identified in these data that would clearly explain the increased Spore formation found in the UK1 *codY* mutant, there were notable differences in the expression of genes indirectly associated with greater sporulation. The transcriptional analyses revealed significant changes in CodY regulation between strains, including increased relative expression of dozens of metabolic genes in UK1 *codY* that are not observed in 630 *codY*. Furthermore, several of the metabolism loci that are upregulated in UK1 *codY* contain differences in their putative CodY boxes compared to 630 *codY* (Table 1), while some are only encoded by one strain (Table 2). The extensive differences in UK1 *codY* and 630 *codY* metabolic gene expression suggest that these strains have altered responses to nutrient limitation, which may affect the ability to initiate or complete spore formation.

### Repression of multiple direct CodY-regulated factors impacts sporulation in strain UK1

**Fig 3:**
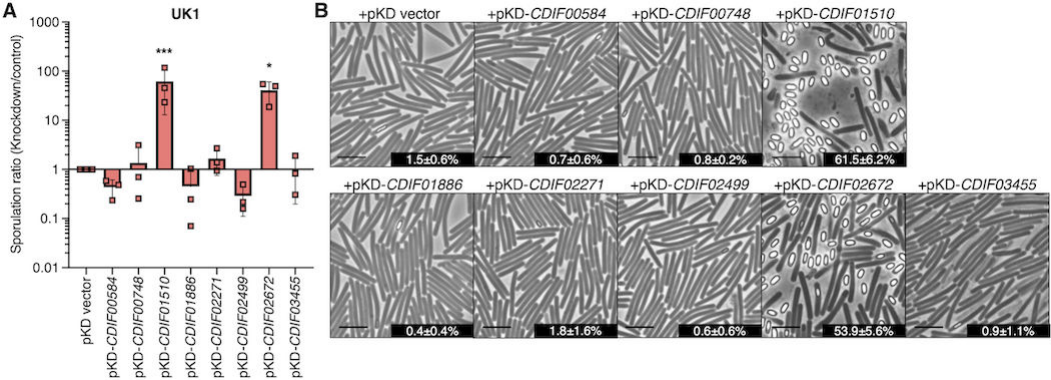
Repression of specific direct CodY-induced factors increases sporulation in UK1. (**A**) Ratio of ethanol-resistant spore formation of strain UK1 expressing CRISPRi knockdown constructs relative to a vector control. UK1 carrying pKD-*CDIF01886* (MC2187)*,* pKD-*CDIF01510* (MC2188)*,* pKD-*CDIF02271* (MC2189)*,* pKD-*CDIF02499* (MC2190)*,* pKD-*CDIF00584* (MC2191)*,* pKD-*CDIF00748* (MC2192)*,* pKD-*CDIF03455* (MC2194)*,* pKD-*CDIF02672* (MC2263)*,* and the pKD vector (MC2186) were assessed for spore formation after 24 h growth on sporulation agar (70:30 with 2 µg/mL thiamphenicol, 1 µg/mL nisin). The means, individual values, and standard deviations of ratios (knockdown/control) for at least three biological replicates are shown. (**B**) Phase-contrast micrographs of the strains in A with sporulation frequencies. Scale bar = 5 µm. The mean, standard deviations, and SEM are shown for three biological replicates. Data were analyzed using a one-way ANOVA, followed by Fisher’s LSD. *P < 0.05 and *** *P* < 0.001.

Given the limited information available on the function of many CodY-regulated factors, we selected an assortment of genes present in both strains that were greatly induced or repressed by CodY for further investigation of their impacts on sporulation (Table 3). To determine which directly regulated CodY-dependent transcripts may impact spore formation, we employed a CRISPR interference (CRISPRi) approach to suppress transcription of target genes (43). The UK1 and UK1 *codY* strains were used for these experiments due to the robust CodY-regulated sporulation phenotype in this background. The UK1 strain was used to evaluate the effects of repressing eight CodY-induced factors, while the UK1 *codY* mutant was used to examine repression of six CodY-repressed factors. Strains were transformed with plasmids containing each CRISPRi sgRNA target expressed from a nisin-inducible promoter and grown on 70:30 agar with 1 µg/mL nisin to assess the impact of transcript repression on sporulation (44, 45). The repression of target genes was examined by qRT-PCR during active growth, which confirmed that the targeted transcripts were reduced in all the strains tested (Fig. S1). The sporulation frequencies of strains carrying each sgRNA target were determined after 24 h, as previously noted, and normalized to the respective parent carrying the vector control (pKD). As shown in Fig. 3, suppression of two of the eight CodY-induced transcripts in strain UK1 resulted in significant increases in sporulation relative to the control. The repression of *CDIF27147_01510*, a gene of unknown function, resulted in a ~40-fold increase in sporulation in strain UK1. The expression of *CDIF27147_01510* was reduced approximately 50-fold in the UK1 *codY* mutant and 20-fold in the 630 *codY* mutant (annotated *CD630_14850* in 630) under sporulation conditions (Table S3). The *CD630_14850* gene is controlled by the iron-responsive regulator, Fur, and induced by cysteine, suggesting it is involved in metabolism (46, 47). Similarly, knockdown of the *CDIF27147_02672* transcript led to ~35-fold greater sporulation in UK1 (Fig. 3). Expression of *CDIF27147_02672* was decreased fourfold in the UK1 *codY* mutant and approximately threefold in the 630 *codY* mutant during sporulation (Table S3). *CDIF27147_02672* is part of a dicistronic operon encoding a pH-dependent transcriptional regulator and transporter we recently characterized (*smrRT; CD630_25050-25060*) that contributes to macrolide and lincosamide resistance (48). SmrR represses expression of the *smrT* transporter, which reduces sporulation and toxin production (48). Expression of SmrRT and CDIF27147_01510 does not appear to directly link to Spo0A activity based on known interactions (42) but more likely support cellular homeostasis through pH or nutritional adaptations, respectively.

**TABLE 3:** CodY-regulated genes of UK1 selected for knockdown

Direct CodY-induced targets				
Predicted CodY target	Predicted CodY Box	∆*codY*/WT	Name	Putative function
CDIF27147_01886	AATTTTAAAAAAATT AATTTTTTGAAAAAA CATTTTCCTAATATT	0.02		Unknown
CDIF27147_01510	CATTATCAGAAAAAT	0.022		Unknown
CDIF27147_02271	AGTTTTTGAAAAATT	0.04–0.04		Transcription regulation
CDIF27147_02499	AAATATCAAAAACTT	0.12		Transcription regulator
CDIF27147_00584	AATATGCAGAAAATG AATTTTCTATAAATA AAAGTTCTGAAAATA AATTATGTGAAAATA	0.18		Transcription antiterminator
CDIF27147_00748	ATATTTCATAAAATT	0.19	*blaI*	Transcription regulator
CDIF27147_03455	AACTTAATGAAAACT AATATTGACAAAATA AATATCCAGAAATAT	0.22–0.24	*spo0E*	Sporulation initiation
CDIF27147_02672	ATTTTTCAAAAATTT	0.24–0.30	*smrR*	Transcription regulator
Direct CodY-repressed targets				
CDIF27147_02081	AATCTTCAAAAAATA	248.6–376.2		Unknown
CDIF27147_00252	AATCTTAATAAACTT	267.7		Unknown
CDIF27147_01772	AAATTTATGAATATT	65.9		Unknown
CDIF27147_02803	GATTTTTAGAAGATT	47.4		Unknown
CDIF27147_01821	AAATCTCAGAAAGTT	42.3		Metabolism
CDIF27147_03734	ATTCTTATGAAAATA AATGTTAATAAAGTT AATATTTAGAATAAT	41.4		Unknown

**Fig 4:**
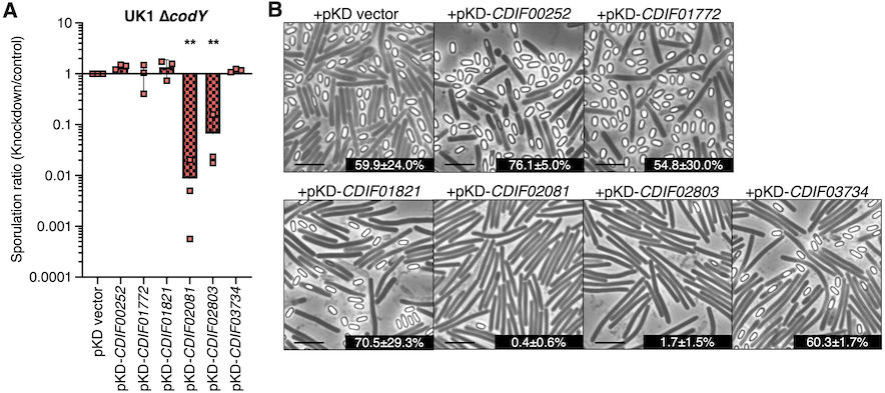
Repression of specific direct CodY-repressed factors reduces sporulation in the UK1 codY mutant. (**A**) Ratio of ethanol-resistant spore formation of strain UK1 ∆*codY* mutant expressing CRISPRi knockdown constructs relative to a vector control. UK1 ∆*codY* carrying pKD-vector (MC2195)*,* pKD-*CDIF00252* (MC2196)*,* pKD-*CDIF01772* (MC2197)*,* pKD-*CDIF01821* (MC2219)*,* pKD-*CDIF02081* (MC2216)*,* pKD-*CDIF02803* (MC2218)*,* and pKD-*CDIF03734* (MC2220) were assessed for spore formation after 24 h growth on sporulation agar (70:30 with 2 µg/mL thiamphenicol, 1 µg/mL nisin). The means, individual values, and standard deviations of ratios (knockdown/control) for at least three biological replicates are shown. (**B**) Phase-contrast micrographs of the strains in A with sporulation frequencies. Scale bar = 5 µm. The mean, standard deviations, and SEM are shown for three biological replicates. Data were analyzed using a one-way ANOVA, followed by Fisher’s LSD. **P < 0.01.

The UK1 *codY* mutant was used to assess repression of six CodY-repressed factors by CRISPRi and examine their effects on sporulation, as outlined above. Of the six genes assessed in UK1 *codY*, suppression of *CDIF27147_02081* and *CDIF27147_02803* dramatically reduced spore formation (Fig. 4). Repression of *CDIF27147_02081* led to a ~150-fold decrease in sporulation, while knockdown of *CDIF27147_02803* resulted in ~35-fold lower spore formation than the control. *CDIF27147_02081* and *CDIF27147_02803* both encode predicted membrane proteins of unknown function that are expressed during sporulation (33, 49–51). *CDIF27147_02081* expression increased 248-fold in the UK1 *codY* mutant during sporulation, but was down 14-fold in the 630 *codY* mutant (*CD630_19280*) (Table S3). Similarly, *CDIF27147_02803* expression increased 47-fold in UK1 *codY* and decreased threefold in 630 *codY* (*CD630_26360*) during sporulation. To determine if the impacts of these transcripts on sporulation are specific to the *codY* mutant, we expressed both knockdown constructs in the wild-type UK1 strain and assessed their effects on sporulation (Fig. S3). We found that repression of either *CDIF27147_02081* or *CDIF27147_02803* led to comparable reduction in sporulation as observed in the *codY* mutant, suggesting that both transcripts are important for spore formation. These results and the contrasting expression profiles for these genes in the UK1 *codY* and 630 *codY* mutants suggest that both factors support robust spore formation, but further investigation is needed to understand their roles in sporulation.

## DISCUSSION

While 630 and UK1 encode identical CodY proteins that can bind to the same target sites, the activity of CodY in these backgrounds may be influenced by many factors that cannot be easily measured. CodY regulation is contingent on the availability of the cofactors GTP and BCAA, which trigger conformational changes in CodY that are necessary for DNA binding (11, 13, 14, 27–29). The availability of GTP and BCAA signals amino acid and energy levels in the cell, which can vary in strains based on their ability to take up nutrients or their capacity to utilize nutrient sources. The UK1 and other 027 isolates grow more poorly than the 630 strain in complete defined minimal media (CDMM), and 027 ribotype isolates demonstrate a narrower metabolic repertoire than 630 and many other strains (52–57). The metabolic range of the 027 isolates relative to other strains may contribute to differences in CodY activity. For example, if BCAA are available to bind CodY, even if other growth-limiting nutrients are unavailable, CodY-DNA binding could persist, restricting adaptation to nutrient limitation and decreasing spore formation (Fig. 1, UK1 24 h). Thus, deletion of *codY* in UK1 could expand metabolite availability through nutrient gene derepression to support sporulation. Our data suggest that at least some of the CodY-regulated genes in UK1 repress sporulation, as indicated by the hypersporulation of the UK1 *codY* mutant, while in the 630∆*erm* strain, only the timing of sporulation is advanced in the absence of *codY* (Fig. 1). Overall, the evidence suggests that nutrient availability differs in these strains, leading to differential CodY regulation of sporulation and metabolic processes.

Our data show that the CodY regulons of the 630*∆erm* and UK1 strains are considerably different (Tables S1 through S3). Additionally, we identified several CodY-dependent genes with putative CodY boxes that differ in these strains (Table 1) and unique CodY-regulated factors present only in one strain (Table 2). Though we were able to identify several factors that are differentially regulated by CodY that have potential CodY-binding sites, further investigation is needed to determine if CodY is the major regulator of these factors and if CodY binds to these boxes. It is also important to note that by limiting our analysis to factors that were differentially expressed in the *codY* mutants by more than threefold, we may have missed some direct CodY-regulated factors that impact sporulation.

Our work demonstrates that multiple factors regulated by CodY can influence sporulation, as illustrated by the phenotypes observed from repression of CodY-regulated factors (Fig. 3 and 4). As CodY regulates hundreds of genes, innumerable effects of global changes in gene expression in the absence of *codY* may contribute to the different sporulation phenotypes in the UK1 and 630∆*erm* strains. The effects of CodY on sporulation may be an indirect result of altering the nutrients available or cellular functions that are necessary for adapting to post-exponential growth. Many of the CodY-dependent factors that are differentially regulated have no identified function in *C. difficile*, and their roles in sporulation are not known. Further characterization of these CodY-regulated factors, especially those that affect sporulation when repressed, could provide targets for preventing spore formation.

## MATERIALS AND METHODS

### Bacterial strains and growth conditions

*C. difficile* strains were cultivated in a Coy anaerobic chamber at 37°C with an atmosphere of 10% H_2_, 5% CO_2_, and 85% N_2_ as previously described (58). *C. difficile* strains grew in BHIS broth with addition of 0.1% of taurocholic acid (TA, Sigma-Aldrich) to induce germination and 0.2% of fructose (D-fructose, Fisher Chemical) to prevent sporulation (59) To maintain plasmids in *C. difficile* strains, 2–10 μg/mL of thiamphenicol was added to cultures. For CRISPRi induction, 1 µg/mL of nisin was added, as needed. *Escherichia coli* strains were cultivated aerobically at 37°C in LB medium (Lennox) with 20 µg/mL of chloramphenicol and/or 100 µg/mL ampicillin (Sigma-Aldrich) for plasmid maintenance. Agar was added at 1.5% for all solid media. *E. coli* was counter-selected post-conjugation with 100 µg/mL of kanamycin.

### Strain and plasmid construction

All plasmids and strains are listed in Table 4. The *C. difficile* strain R20291 027 ribotype genome (GenBank accession no. CP_029423.1) was used as a template for primer construction, and UK1 genomic DNA was used for PCR amplification. To generate sgRNAs, the Benchling CRISPR Guide RNA Design tool was used. sgRNAs were amplified by PCR and cloned into pMC1123 (43, 60). Design details of vector constructions are provided in the supplemental material (Fig. S2).

**TABLE 4:** Bacterial strains and plasmids

Plasmid or strain	Relevant genotype or features	Source, construction, or reference
Strains		
*E. coli*		
DH5α max efficiency	F− Φ80*lac*ZΔM15 Δ(*lac*ZYA-*arg*F) U169 *rec*A1 *end*A1 *hsd*R17 (rk−, mk+) *pho*A *sup*E44 λ*−*thi−1 *gyr*A96 *rel*A1	Invitrogen
HB101	F^-^ *mcrB mrr hsdS20*(r_B_^-^ m_B_*^-^) recA13 leuB6 ara-14 proA2 lacY1 galK2 xyl-5 mtl-1 rpsL20*	Dupuy
*C. difficile*		
630Δ*erm*	Erm^S^ derivative of strain 630, ribotype 012	Minton (61)
UK1	Epidemic isolate, ribotype 027	(62)
LB-CD16	UK1 *codY::ermB*	(63)
MC310	630Δ*erm spo0A::ermB*	(36)
MC364	630Δ*erm codY::ermB*	(13)
MC855	630Δ*erm spo0A::ermB* pMC123	(41)
MC2186	UK1 pMC1123	(48)
MC2187	UK1 pMC1170	This study
MC2188	UK1 pMC1171	This study
MC2189	UK1 pMC1172	This study
MC2190	UK1 pMC1173	This study
MC2191	UK1 pMC1174	This study
MC2192	UK1 pMC1175	This study
MC2194	UK1 pMC1177	This study
MC2195	UK1 *codY::ermB* pMC1123	This study
MC2196	UK1 *codY::ermB* pMC1158	This study
MC2197	UK1 *codY::ermB* pMC1160	This study
MC2216	UK1 *codY::ermB* pMC1156	This study
MC2218	UK1 *codY::ermB* pMC1162	This study
MC2219	UK1 *codY::ermB* pMC1163	This study
MC2220	UK1 *codY::ermB* pMC1164	This study
MC2263	UK1 pMC1178	(48)
MC3087	UK1 pMC1156	This study
MC3088	UK1 pMC1162	This study
Plasmids		
pRK24	Tra^+^, Mob^+^; *bla, tet*	(64)
pIA33	P*_xyl_::dCas9-opt* P*_gdh_*::sgRNA-*rfp catP*	(43)
pMC123	*E. coli- C. difficile* shuttle vector*, bla, catP*	(45)
pMC404	pMC123 with *catP* replaced by *aad9*	(65)
pMC1123	P*_cprA_::dCas9-opt* P*_gdh_*::sgRNA*-neg catP*; (pKD)	(60)
pMC1156	P*_cprA_::dCas9-opt* P*_gdh_*::sgRNA*-CDIF27147_02081 catP*	This study
pMC1158	P*_cprA_::dCas9-opt* P*_gdh_*::sgRNA*-CDIF27147_00252 catP*	This study
pMC1160	P*_cprA_::dCas9-opt* P*_gdh_*::sgRNA*-CDIF27147_01772 catP*	This study
pMC1162	P*_cprA_::dCas9-opt* P*_gdh_*::sgRNA*-CDIF27147_02803 catP*	This study
pMC1163	P*_cprA_::dCas9-opt* P*_gdh_*::sgRNA*-CDIF27147_01821 catP*	This study
pMC1164	P*_cprA_::dCas9-opt* P*_gdh_*::sgRNA*-CDIF27147_03734 catP*	This study
pMC1170	P*_cprA_::dCas9-opt* P*_gdh_*::sgRNA*-CDIF27147_01886 catP*	This study
pMC1171	P*_cprA_::dCas9-opt* P*_gdh_*::sgRNA*-CDIF27147_01510 catP*	This study
pMC1172	P*_cprA_::dCas9-opt* P*_gdh_*::sgRNA*-CDIF27147_02271 catP*	This study
pMC1173	P*_cprA_::dCas9-opt* P*_gdh_*::sgRNA*-CDIF27147_02499 catP*	This study
pMC1174	P*_cprA_::dCas9-opt* P*_gdh_*::sgRNA*-CDIF27147_00584 catP*	This study
pMC1175	P*_cprA_::dCas9-opt* P*_gdh_*::sgRNA*-CDIF27147_00748 catP*	This study
pMC1177	P*_cprA_::dCas9-opt* P*_gdh_*::sgRNA*-CDIF27147_03455 catP*	This study
pMC1178	P*_cprA_::dCas9-opt* P*_gdh_*::sgRNA*-CDIF27147_02672 catP*	(48)

### Sporulation assays

Sporulation assays were carried out as previously described (66, 67). In short, *C. difficile* cultures at mid-exponential phase (OD_600_ ~0.5) were plated on 70:30 agar supplemented with 2 μg/mL of thiamphenicol and 1 μg/mL of nisin as needed. After 6 (H_6_), 12 (H_12_), and 24 h (H_24_) of growth, ethanol-resistant sporulation assays were performed as previously described (67). Sporulation frequencies were calculated by dividing the number of spores by the total quantity of cells (spores + vegetative). A *spo0A* mutant was used as a negative sporulation control. For statistical analysis, GraphPad Prism v10.4.1 was used as stated in the figure legends.

### Phase-contrast microscopy

Phase-contrast microscopy was performed at H_6_, H_12_, and H_24_ as specified in the figure legends using cells grown on 70:30 sporulation agar, as previously described (60).

### RNA sequencing (RNA-seq) analysis

*C. difficile* strains were grown on 70:30 agar for 6 h, and cells were scraped, suspended into 1:1:2 ethanol-acetone-water solution, and stored at −70°C prior to processing. RNA was extracted and treated with DNase I (Ambion), as previously described (14, 36). RNA libraries were prepared and processed by the Microbial Genomics Sequencing Center (MiGS; Pittsburgh, PA), as previously described. RNA-seq reads were mapped to the respective reference genome (630; NC_009089.1 and R20291; CP_029423.1) using Geneious Prime v2022.2.2. Expression levels of transcripts were calculated and compared using DESeq2 (68) RNA-seq raw sequence reads were deposited to the NCBI Sequence Read Archive (SRA) as BioProject PRJNA1263881.

### Identification of CodY boxes

Potential CodY boxes were found in the 630 and R20291 genomes from previously published sites, in addition to *in silico* identification (14, 37). The *C. difficile* strain 630 and R20291 genomes (630, NC_009089.1; R20291, CP_029423.1) were screened for the global CodY AATTTTCWGAAAATT consensus sequence containing up to four mismatches using a combination of FIMO MEME and Benchling software (14, 38).

### Quantitative reverse transcription PCR analysis (qRT-PCR)

*C. difficile* strains were grown on 70:30 agar for 6 h, suspended in 1:1:2 ethanol-acetone-water solution, and stored at −70°C. RNA extraction, treatment with DNase I (Ambion), and cDNA synthesis using random hexamers (Bioline) were performed as previously described (14, 36). qRT-PCR was conducted on a Roche LightCycler 96 instrument from 50 ng of cDNA in technical triplicates using Bioline SensiFast SYBR & Fluorescein Mix, with primers shown in Table 5. Expression was normalized to the internal control transcript, *rpoC*, and analyzed using the ∆∆C_t_ method for relative quantification (69). GraphPad Prism v10.4.1 was used as mentioned in the figure legends for statistical analysis.

**TABLE 5:** Oligonucleotides

Primer	Sequence (5′→3′)	Use/locus tag/reference
oMC44	CTAGCTGCTCCTATGTCTCACATC	Forward primer for *rpoC* qPCR (45)
oMC45	CCAGTCTCTCCTGGATCAACTA	Reverse primer for *rpoC* qPCR (45)
oMC2618	GATTATTATGGCGAACAATGAATTAGAAG	Forward primer for *spo0E* qPCR
oMC2619	AAATATTTCTGGATATTCTATGTATGTATTTATCT	Reverse primer for *spo0E* qPCR
oMC2362	AGTTAAACAGAAAGATAATTGCTGTATGG	Forward primer for *smrR* qPCR (48)
oMC2363	ACTTGTAGCCTTACGTTGTTCTTC	Reverse primer for *smrR* qPCR (48)
oMC3088	TTGCAATAAAGTGTGCTATAATTAAACTGTAAATGGCCA	Forward primer to Gibson assemble CRISPRi sgRNAs into pMC1123 (44, 48)
oMC3089	CCTTTTTCTATTTAAAGTTTTATTAAAACTTATAGGATCCGCGGCCGC	Reverse primer to Gibson assemble CRISPRi sgRNAs into pMC1123 (44, 48)
oMC3101	AATTAAACTGTAAATGGCCAAATAATTCCTCACTATCAAGGTTTTAGAGCTAGAAATAGC	Forward primer for sgRNA-*CDIF27147_02081* amplification
oMC3103	AATTAAACTGTAAATGGCCAGAAGAATTACTAAAACTGAGGTTTTAGAGCTAGAAATAGC	Forward primer for sgRNA-*CDIF27147_00252* amplification
oMC3105	AATTAAACTGTAAATGGCCAAATAGTATATTAAAACATAAGTTTTAGAGCTAGAAATAGC	Forward primer for sgRNA-*CDIF27147_01772* amplification
oMC3108	AATTAAACTGTAAATGGCCAACAACAGTTTCAAGGTCTTGGTTTTAGAGCTAGAAATAGC	Forward primer for sgRNA-*CDIF27147_02803* amplification
oMC3109	AATTAAACTGTAAATGGCCATTGACTTGGATAGTACCAAGGTTTTAGAGCTAGAAATAGC	Forward primer for sgRNA-*CDIF27147_01821* amplification
oMC3110	AATTAAACTGTAAATGGCCAATATTTTTGTAAGGATGCAAGTTTTAGAGCTAGAAATAGC	Forward primer for sgRNA-*CDIF27147_03734* amplification
oMC3131	AATTAAACTGTAAATGGCCATCTTGAAGGTGGTAAAATGGGTTTTAGAGCTAGAAATAGC	Forward primer for sgRNA-*CDIF27147_01886* amplification
oMC3132	AATTAAACTGTAAATGGCCATGGTGACACAAAACAATCCGGTTTTAGAGCTAGAAATAGC	Forward primer for sgRNA-*CDIF27147_01510* amplification
oMC3133	AATTAAACTGTAAATGGCCAGGTATACAAAAGTTTAAGCAGTTTTAGAGCTAGAAATAGC	Forward primer for sgRNA-*CDIF27147_02271* amplification
oMC3134	AATTAAACTGTAAATGGCCAAAAAACGTACCTAAAACTGTGTTTTAGAGCTAGAAATAGC	Forward primer for sgRNA-*CDIF27147_02499* amplification
oMC3135	AATTAAACTGTAAATGGCCAAAAAACGTACCTAAAACTGTGTTTTAGAGCTAGAAATAGC	Forward primer for sgRNA-*CDIF27147_00584* amplification
oMC3136	AATTAAACTGTAAATGGCCAATATCTTACTTATTGAAGAGGTTTTAGAGCTAGAAATAGC	Forward primer for sgRNA-*CDIF27147_00748* amplification
oMC3138	AATTAAACTGTAAATGGCCAAAATGAGATTGAAGCAGTTAGTTTTAGAGCTAGAAATAGC	Forward primer for sgRNA-*CDIF27147_03455* amplification
oMC3139	AATTAAACTGTAAATGGCCAATAAAAAAATTATACGTCGAGTTTTAGAGCTAGAAATAGC	Forward primer for sgRNA-*CDIF27147_02672* amplification (48)
oMC3235	TTTCTTAATTATGGCTATGGCAGTT	Forward primer for *CDIF27147_01886* qPCR
oMC3236	ATAAAGGCTTCATAAATACAGCGAA	Reverse primer for *CDIF27147_01886* qPCR
oMC3237	TTGTGTCACCATAAACTTTCCAATA	Forward primer for *CDIF27147_01510* qPCR
oMC3238	AGAGTGATGTTTTTCCTGATGAAAT	Reverse primer for *CDIF27147_01510* qPCR
oMC3239	AACCATCTAAGTTTGGCATCATTAT	Forward primer for *CDIF27147_02271* qPCR
oMC3240	TTTAAGTGCAGAAGGTTATCAAGTT	Reverse primer for *CDIF27147_02271* qPCR
oMC3241	AAATGACTTGGCTTCAACAATATTG	Forward primer for *CDIF27147_02499* qPCR
oMC3242	AGTAATTGCACGTTCTAATGGTATT	Reverse primer for *CDIF27147_02499* qPCR
oMC3243	CAAAGTACGGCCAATTAAATTTTCT	Forward primer for *CDIF27147_00584* qPCR
oMC3244	AATTGCTAACCATTCATCTCTTGAT	Reverse primer for *CDIF27147_00584* qPCR
oMC3245	AAAGTTGCCACAATCAGAATTAAAG	Forward primer for *CDIF27147_00748* qPCR
oMC3246	CGTTTGTTTCCATTGATATTTTTGC	Reverse primer for *CDIF27147_00748* qPCR
oMC3249	AGGTTTGACAAGGCTTTCTAAAATA	Forward primer for *CDIF27147_00252* qPCR
oMC3250	TCAACCATATTTCCAGCATTTGATA	Reverse primer for *CDIF27147_00252* qPCR
oMC3251	CTGCTGTTAATTCAAAATGGAGTTT	Forward primer for *CDIF27147_01772* qPCR
oMC3252	ATCTTATCATTTTTATCCTCTCCATT	Reverse primer for *CDIF27147_01772* qPCR
oMC3394	CACAATAGCTAAAATTGTGCAATGA	Forward primer for *CDIF27147_02803* qPCR
oMC3395	TGCTTATGTTGAAGAAATAGCATCT	Reverse primer for *CDIF27147_02803* qPCR
oMC3396	AATGATTTTATTTGGACTTGGAGGT	Forward primer for *CDIF27147_01821* qPCR
oMC3397	AATTGCTATTGCTGTTAGAGAATCA	Reverse primer for *CDIF27147_01821* qPCR
oMC3398	AGTTGTACCCTCAAAAATATCCATT	Forward primer for *CDIF27147_03734* qPCR
oMC3399	ATTTTGTGTTGGATTTTTGGTTCTT	Reverse primer for *CDIF27147_03734* qPCR
oMC3488	ACGTTACTATTATTGATAATCTTCACTTATATG	Forward primer for *CDIF27147_02081* qPCR
oMC3489	AGATTATAGTACAATAATATAGAAAATTGACACT	Reverse primer for *CDIF27147_02081* qPCR
4084	AACTTATAGGATCCGCGGCCGCTAGTCAGACATCATGCTGATCTAGA	Reverse primer for sgRNAs with NotI site for cloning into pIA33 (43)

## Supplementary Material

Reviewer comments

## SUPPLEMENTAL MATERIAL

Supplemental figures spectrum.01706-25-s0001.pdf Figures S1 to S3.

Table S1 spectrum.01706-25-s0002.xlsx RNA-Seq analysis of 630*erm codY* at H6 on 70:30 sporulation agar.

Table S2 spectrum.01706-25-s0003.xlsx RNA-Seq analysis of UK1 *codY* at H6 on 70:30 sporulation agar.

Table S3 spectrum.01706-25-s0004.xlsx Differentially expressed genes with predicted CodY boxes in 630 *codY* and UK1 *codY*.

OPEN PEER REVIEW reviewer-comments.pdf An accounting of the reviewer comments and feedback.

ASM does not own the copyrights to Supplemental Material that may be linked to, or accessed through, an article. The authors have granted ASM a non-exclusive, world-wide license to publish the Supplemental Material files. Please contact the corresponding author directly for reuse.
