# Supplementary material for: Deciphering strain differences in codY regulation of Clostridioides difficiles sporulation
Source: Microbiol Spectr. 2025 Nov 17;14(1):e01706-25. doi: 10.1128/spectrum.01706-25 (PMC12772354; doi:10.1128/spectrum.01706-25)
Supplement: Supplemental figures — Figures S1 to S3. [file spectrum.01706-25-s0001.pdf]

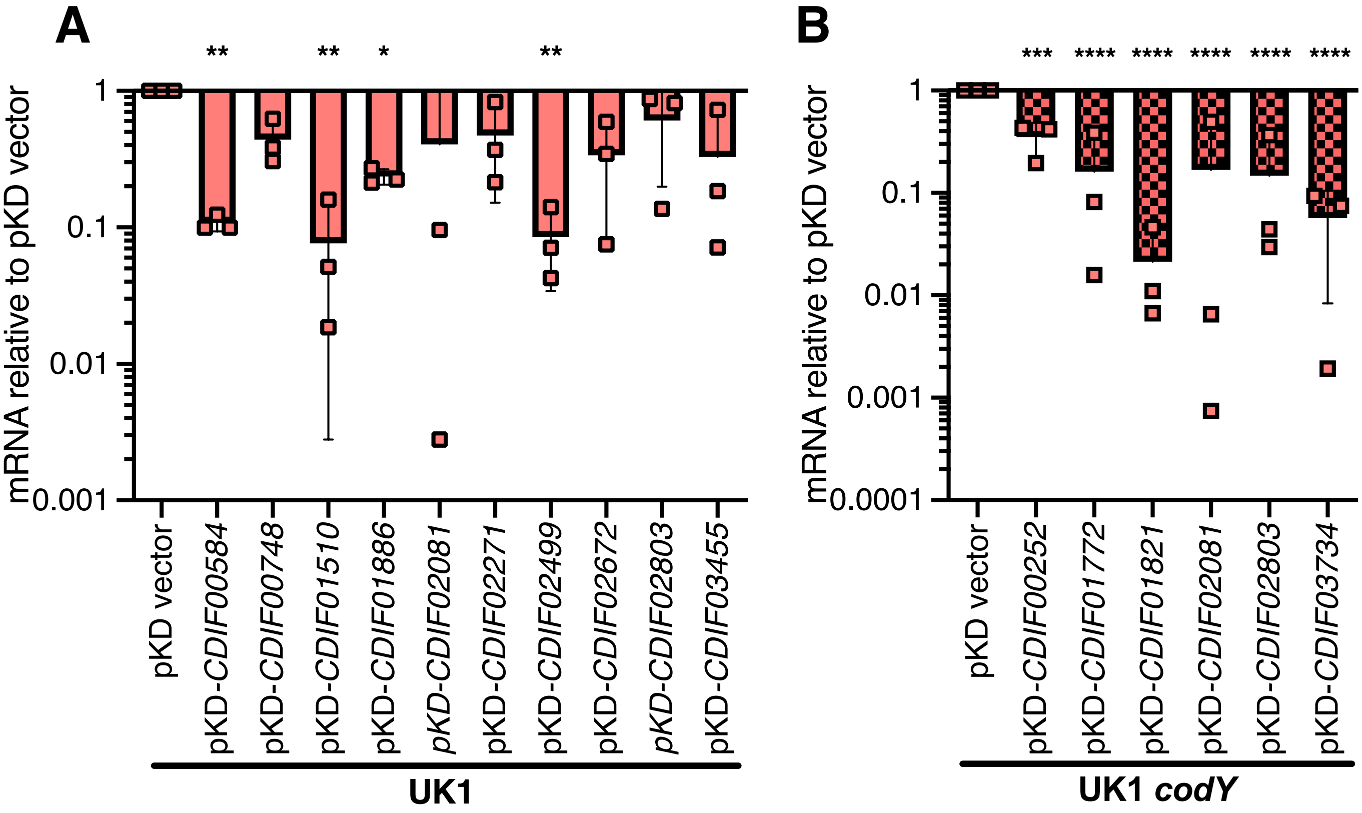

Figure S1. CRISPRi constructs repress expression of target genes. qRT-PCR analysis of gene expression for **A)** UK1 strains expressing CRISPRi knockdown constructs pKD-*CDIF00584* (MC2191), pKD-*CDIF00748* (MC2192), pKD-*CDIF01510* (MC2188), pKD-*CDIF01886* (MC2187), pKD-*CDIF02081* (MC3087), pKD-*CDIF02271* (MC2189), pKD-*CDIF02499* (MC2190), pKD-*CDIF02672* (MC2263), pKD-*CDIF02803* (MC3088), pKD-*CDIF03455* (MC2194), relative to the pKD vector control strain (MC2186) and **B)** UK1 Δ*codY* carrying pKD-*CDIF00252* (MC2196), pKD-*CDIF01772* (MC2197), pKD-*CDIF01821* (MC2219), pKD-*CDIF02081* (MC2216), pKD-*CDIF02803* (MC2218), pKD-*CDIF03734* (MC2220), relative to the pKD-vector control strain (MC2195). Samples were harvested after 6 h of growth on sporulation agar (70:30 with 2 μg/ml thiamphenicol, 1 μg/ml nisin). The means and individual values for three biological replicates are shown. Data were analyzed using a one-way ANOVA followed by Dunnett's multiple comparison test. *P<0.05, ** P<0.01, *** P<0.001, **** P<0.0001.

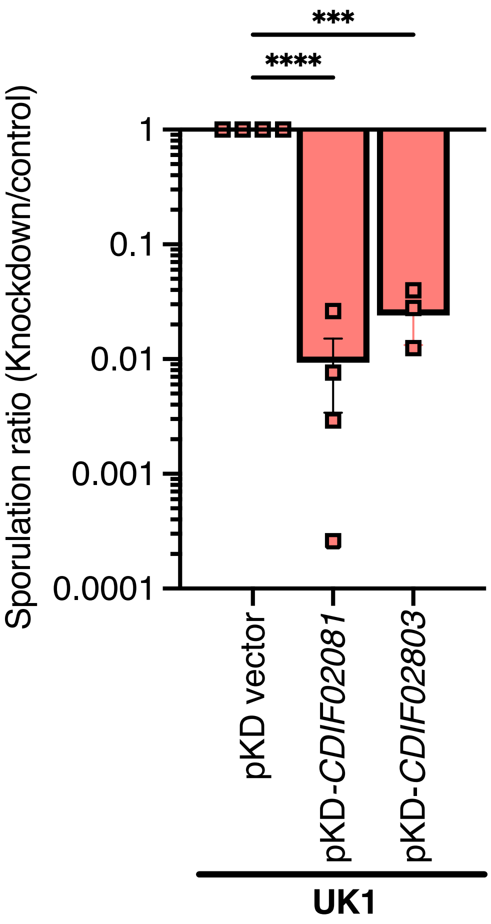

Figure S2. Repression of CodY-repressed factors decreases sporulation in UK1. Ratio of ethanol-resistant spore formation of strain UK1 expressing CRISPRi knockdown constructs, relative to a vector control. UK1 pKD-*CDIF02081* (MC3087), pKD-*CDIF02803* (MC3088), or the pKD vector (MC2186) were assessed for spore formation after 24 h growth on sporulation agar (70:30 with 2 μg/ml thiamphenicol, 1 μg/ml nisin). The means, individual values, and standard deviations of ratios (Knockdown/control) for at least three biological replicates are shown. Data were analyzed using a one-way ANOVA followed by Dunnett's multiple comparisons test. *P<0.05, *** P<0.001.

Figure S3 – DNA cloning and vector details

pMC1156: A 140 bp PCR product containing sgRNA-*CDIF02081* was created using primers 4084 and oMC3101 and was amplified again using primers oMC3088 and oMC2089, which contain homology to pMC1123. The resulting product was Gibson assembled into pMC1123 via *MscI* and *NotI* sites.

pMC1158: A 140 bp PCR product containing sgRNA-*CDIF00252* was created using primers 4084 and oMC3103 and was amplified again using primers oMC3088 and oMC2089, which contain homology to pMC1123. The resulting product was Gibson assembled into pMC1123 via *MscI* and *NotI* sites.

pMC1160: A 140 bp PCR product containing sgRNA-*CDIF01772* was created using primers 4084 and oMC3105 and was amplified again using primers oMC3088 and oMC2089, which contain homology to pMC1123. The resulting product was Gibson assembled into pMC1123 via *MscI* and *NotI* sites.

pMC1162: A 140 bp PCR product containing sgRNA-*CDIF02803* was created using primers 4084 and oMC3108 and was amplified again using primers oMC3088 and oMC2089, which contain homology to pMC1123. The resulting product was Gibson assembled into pMC1123 via *MscI* and *NotI* sites.

pMC1163: A 140 bp PCR product containing sgRNA-*CDIF01821* was created using primers 4084 and oMC3109 and was amplified again using primers oMC3088 and oMC2089, which contain homology to pMC1123. The resulting product was Gibson assembled into pMC1123 via *MscI* and *NotI* sites.

pMC1164: A 140 bp PCR product containing sgRNA-*CDIF03734* was created using primers 4084 and oMC31110 and was amplified again using primers oMC3088 and oMC2089, which contain homology to pMC1123. The resulting product was Gibson assembled into pMC1123 via *MscI* and *NotI* sites.

pMC1170: A 140 bp PCR product containing sgRNA-*CDIF01886* was created using primers 4084 and oMC3131 and was amplified again using primers oMC3088 and oMC2089, which contain homology to pMC1123. The resulting product was Gibson assembled into pMC1123 via *MscI* and *NotI* sites.

pMC1171: A 140 bp PCR product containing sgRNA-*CDIF01510* was created using primers 4084 and oMC3132 and was amplified again using primers oMC3088 and oMC2089, which contain homology to pMC1123. The resulting product was Gibson assembled into pMC1123 via *MscI* and *NotI* sites.

pMC1172: A 140 bp PCR product containing sgRNA-*CDIF02271* was created using primers 4084 and oMC3133 and was amplified again using primers oMC3088 and oMC2089, which contain homology to pMC1123. The resulting product was Gibson assembled into pMC1123 via *MscI* and *NotI* sites.

pMC1173: A 140 bp PCR product containing sgRNA-*CDIF02499* was created using primers 4084 and oMC3134 and was amplified again using primers oMC3088 and oMC2089, which contain homology to pMC1123. The resulting product was Gibson assembled into pMC1123 via *MscI* and *NotI* sites.

pMC1174: A 140 bp PCR product containing sgRNA-*CDIF0584* was created using primers 4084 and oMC3135 and was amplified again using primers oMC3088 and oMC2089, which contain homology to pMC1123. The resulting product was Gibson assembled into pMC1123 via *MscI* and *NotI* sites.

pMC1175: A 140 bp PCR product containing sgRNA-*CDIF00748* was created using primers 4084 and oMC3136 and was amplified again using primers oMC3088 and oMC2089, which contain homology to pMC1123. The resulting product was Gibson assembled into pMC1123 via *MscI* and *NotI* sites.

pMC1177: A 140 bp PCR product containing sgRNA-*CDIF03455* was created using primers 4084 and oMC3138 and was amplified again using primers oMC3088 and oMC2089, which contain homology to pMC1123. The resulting product was Gibson assembled into pMC1123 via *MscI* and *NotI* sites.
